# Ocular Syphilis Presenting As Non-arteritic Anterior Ischemic Optic Neuropathy

**DOI:** 10.7759/cureus.16694

**Published:** 2021-07-28

**Authors:** Moayad M Alqurashi, Maha Badr, Abdullah Bukhari

**Affiliations:** 1 Division of Adult Infectious Diseases, Deprtment of Medicine, Prince Sultan Military Medical City, Riyadh, SAU; 2 Department of Ophthalmology, Prince Sultan Military Medical City, Riyadh, SAU; 3 Department of Medicine, Faculty of Medicine, Imam Mohammed Ibn Saud Islamic University, Riyadh, SAU

**Keywords:** syphilis, ocular syphilis, neurosyphilis, na-aion, saudi arabia, onychomadesis, secondary syphilis, bacterial sexually transmitted infections, ischemic optic neuropathy

## Abstract

Syphilis is a sexually transmitted disease caused by the spirochetal bacteria *Treponema pallidum*. It can cross the blood-brain barrier within days of the infection, causing neurosyphilis and ocular syphilis at any stage of the disease. Ocular syphilis can manifest in any part of the eye but usually as posterior uveitis and pan-uveitis or various types of inflammatory or immune-mediated optic neuritis. Misdiagnosing ocular syphilis as a non-infectious disease has been reported even when seen by ophthalmologists due to the wide variety of possible presentations. In this case report, we describe a case of ocular syphilis that presented with a non-arteritic anterior ischemic optic neuropathy (NA-AION), which to our knowledge, has not been described before in the literature.

## Introduction

Syphilis is a sexually transmitted disease that spreads via direct contact to an infected mucocutaneous lesion or vertically from mother to child [1]. The causative organism (*Treponema pallidum* subspecies *pallidum*) is one of four subspecies of the same genus that none of which are sexually transmitted or have such a wide geographical spread [1]. The organism was first identified in 1905 by Schaudinn and Hoffman, but the disease was described with different names much earlier [2]. The organism resists being cultured *in vitro* and does not have an animal reservoir which complicates studying it [1].

Worldwide, syphilis cases are increasing dramatically, and local data from the Middle East are scarce [3]. A retrospective study from Saudi Arabia comparing two datasets from 1995 to 1999 with data from 2005 to 2012 rates of reported cases of syphilis dropped to 2.56% from 8.7%, which is an underestimation partially due to social stigmatization of sexually transmitted infections in general and or underreporting [4].

The clinical course of the disease is usually described as primary, secondary, latent, or tertiary [1]. These stages can also be described as early versus late syphilis with a cut-off of one year (American and European guidelines) vs. two years (WHO guidelines) since the possible date of infection [1]. Syphilis has a wide variety of clinical presentations and any tissues can be invaded by *T. pallidum* [1]. It can cross the blood-brain barrier, causing neurosyphilis and ocular syphilis [1,3]. Ocular syphilis can manifest in any part of the eye but usually as posterior uveitis and pan-uveitis [3]. Other manifestations include retinitis, interstitial keratitis, placoid chorioretinitis, papillitis, retinal vasculitis, cranial nerve palsies, or different forms of optic neuritis (ON) [5].

Here we present a case of ocular syphilis that presented with the unilateral visual loss associated with a widespread skin rash that was diagnosed as non-arteritic anterior ischemic optic neuropathy (NA-AION) after excluding other diagnoses.

## Case presentation

**Figure 1:**
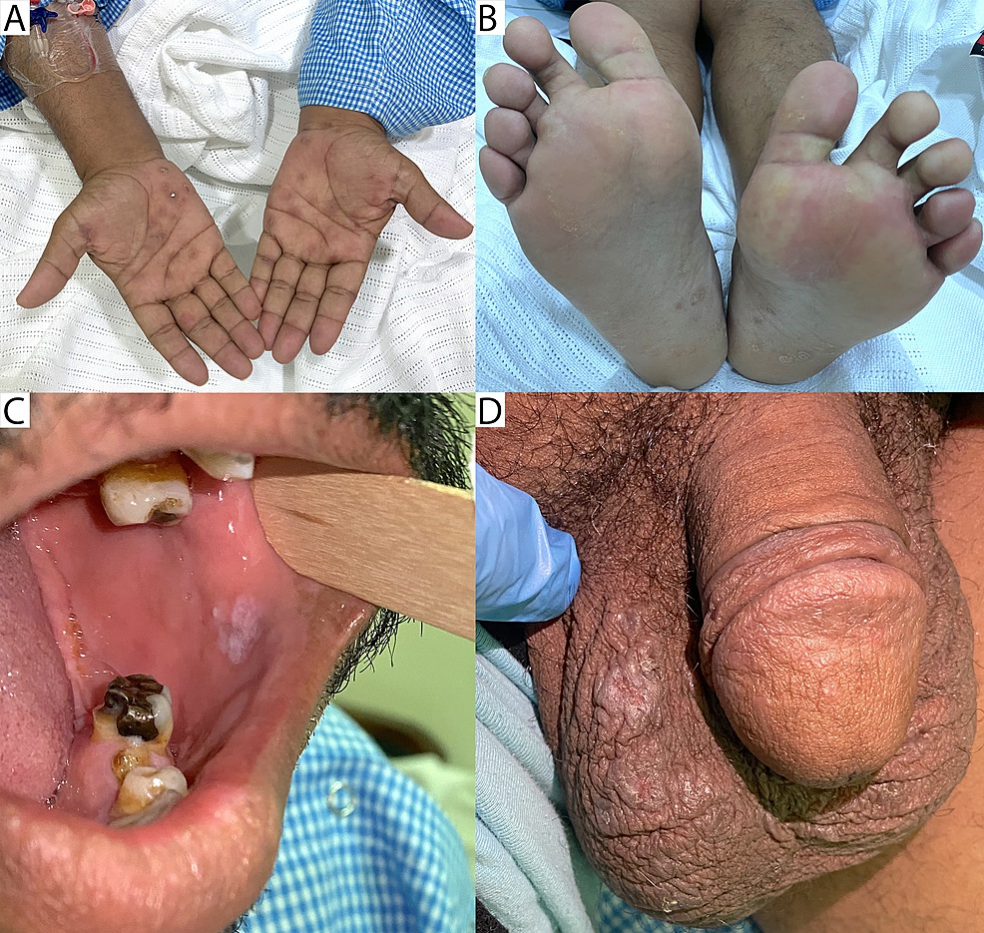
Clinical signs at presentation (A) A maculopapular rash on both palms. (B) A maculopapular rash with small scales on both soles. (C) A mucus patch on the inside of the left cheek. (D) A small ulcer over the right testicle.

**Figure 2:**
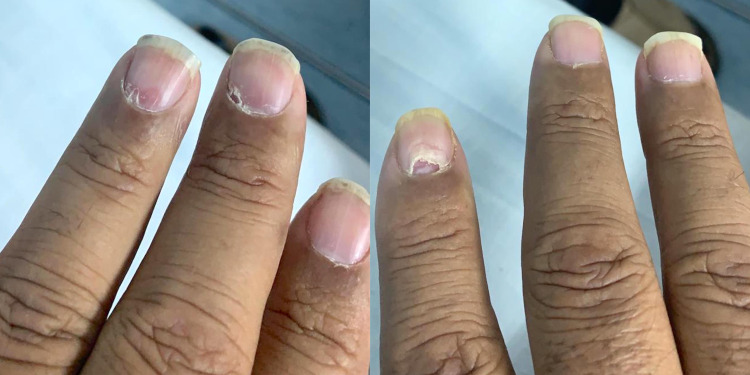
Onychomadesis on some nails of both hands

A 50-year-old obese ex-smoker male suffers from chronic uncontrolled type two diabetes mellitus and dyslipidemia for more than 10 years. He presented to the emergency department (ED) with a progressive, painless right-eye decrease in vision over the previous two weeks. He primarily complained of worsening central vision and color vision of the affected eye. At presentation, he had a bilateral faint maculopapular rash covering both palms and soles (Figures 1A, 1B) and had a mucus patch on the inside of his left cheek (Figure 1C) and a genital skin rash over his right testicle (Figure 1D). He also had onychomadesis in some nails on both hands (Figure 2). He denied any other relevant symptoms like fever, headaches, or red eyes. He reported a single event of unprotected casual sexual encounter during a trip to Indonesia 11 months before his ED presentation.

On physical examination, his vital signs were within normal limits. A complete neurological examination was unremarkable; however, an ophthalmological examination revealed some interesting findings that are summarized in Table 1.

**Table 1:** Eye examination and ancillary testing in both episodes *Confrontational visual field **Automated perimetry Abbreviations: OS: Oculus sinister (left eye), OD: Oculus dextrus (right eye), RAPD: Relative afferent pupillary defect, NPDR: Non-proliferative diabetic retinopathy, OCT: Optical coherence tomography, SC: Uncorrected.

Eye examination and ancillary testing of the first hospital visit (December 2019)		
Exam	OD	OS
Pupil reaction to light	Sluggish	Normal reaction
RAPD	1+	No defect
Cornea and anterior chamber	Clear, deep, and quite	Clear, deep, and quite
Fundus exam	Mild NPDR, optic disc swelling with splinter hemorrhages	Mild NPDR, disc at risk
Visual acuity SC	6/36	6/9
Color vision	1/21	21/21
Visual field*	Superior and Inferior arcuate scotoma	Normal
Extraocular muscles	Intact	Intact
OCT	Normal	Normal
Eye examination and ancillary testing of the second hospital visit (August 2020)		
Exam	OD	OS
Pupil reaction to light	Sluggish	Sluggish
Cornea and anterior chamber	Clear, deep, and quite	Clear, deep, and quite
Fundus exam	Mild NPDR, mild optic disc pallor	Mild NPDR, marked optic disc pallor
Visual acuity SC	6/30	Counting fingers at 1 meter
Color vision	1/21	1/21
Visual field**	Superior arcuate and Inferior altitudinal defect	Inferior altitudinal defect
Extraocular muscles	Intact	Intact
OCT	Normal	Normal

**Figure 3:**
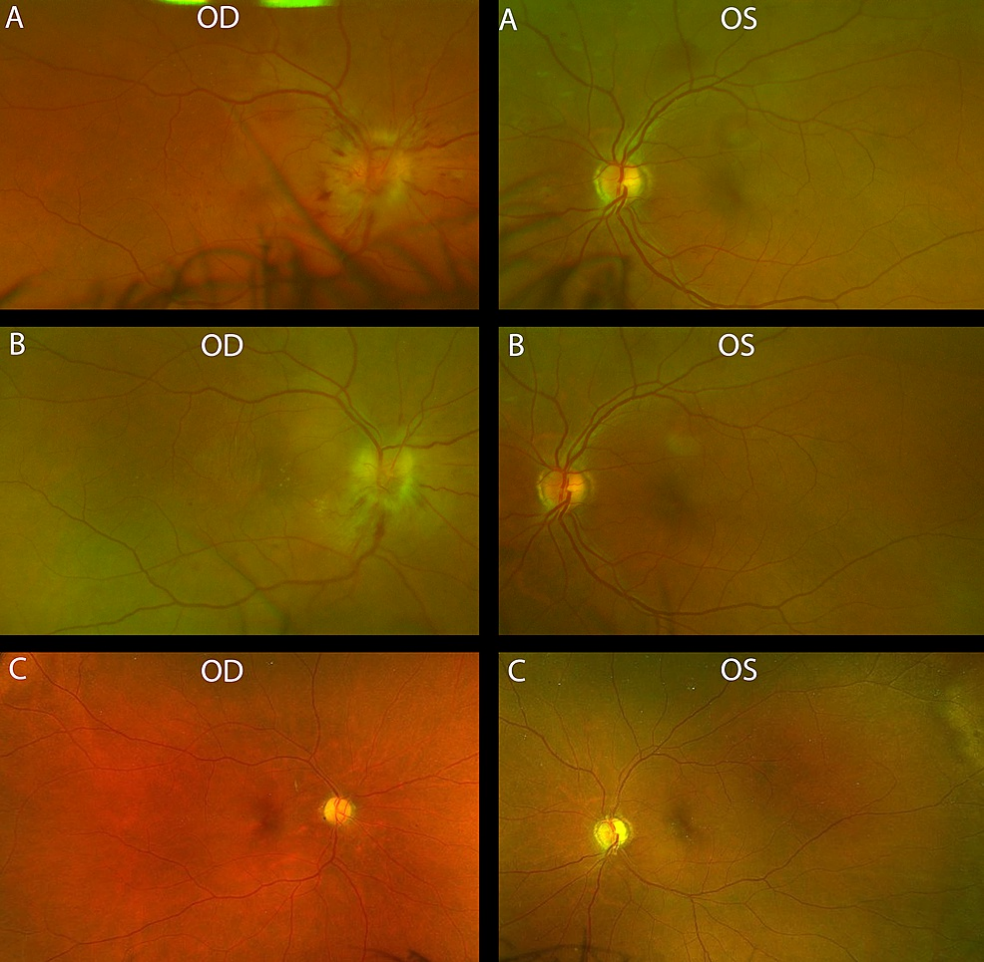
Fundus exam of both eyes Fundus exam on the (A) first episode showing right eye disc swelling with splinter hemorrhages and disc at risk in the left eye, (B) follow-up at three months showing pale swollen disc on the right eye and disc at risk on the left eye, (C) follow-up at one year showing bilateral atrophied discs without dilation. Abbreviations: OS: Oculus sinister (left eye), OD: Oculus dextrus (right eye)

There were no signs of uveitis or any inflammation. The fundus examination is shown in Figure 3A. Fundus fluorescein angiography (FFA) showed leakage in the right optic disc only. The patient was started on Ceftriaxone 2 g IV once daily for four days till benzylpenicillin became available, for which he received 2.4 g (4 megaunits) IV every four hours for 10 days. Adjunctive therapy with high-dose steroids was started with tapering over two weeks with adjustments of his diabetic control medications. One month after discharge, he reported no improvement of his vision but complete resolution of the rashes all over his body. At three months, there was a slight improvement in his visual acuity of the right eye, but dilated fundus examination showed a pale right optic disc (Figure 3B). Five months after discharge, he did suffer another episode of painless loss of vision of the left eye that was sudden and not associated with other signs or symptoms. At a one-year follow-up, there were no significant changes in his ophthalmological examination (Figure 3C).

Brain CT and MRI revealed no enhancement in the post-contrast images, including the spine. The optic nerves were unremarkable bilaterally with no enhancement in the fat-suppressed images at any part of the nerve. Visual evoked potentials (VEP) showed absent NPN complexes in the right eye while the left eye had deformed NPN complexes with low amplitude and P100 latency at 109 ms. The cerebrospinal fluid (CSF) analysis and other investigations were suggestive of secondary syphilis with ocular/neurosyphilis, as shown in Table 2. A biopsy was taken from the rash on his palm showing hypergranulosis with band-like inflammation and saw-tooth rete ridges, supporting the diagnosis of secondary syphilis.

**Table 2:** Laboratory investigations and CSF analyses of the first and second hospital visits Abbreviations: WBC: White blood cells, PLT: Platelets, ALT: Alanine transaminase, ALP: Alkaline phosphatase, TSH: Thyroid-stimulating hormone, LDL: Low-density lipoprotein, ACE: Angiotensin-converting enzyme, ANA: Antinuclear antibody, MOG: Myelin oligodendrocyte, AQP4: Aquaporin 4, ESR: Erythrocyte sedimentation rate, CRP: C-reactive protein, HBV: Hepatitis B Virus, HCV: Hepatitis C Virus, HIV: Human immunodeficiency virus, TP: Treponema pallidum, CLIA: Chemiluminescence immunoassay, IgM: Immunoglobulin M, EIA: Enzyme Immunoassay, CSF: Cerebrospinal fluid, RBC: Red blood cells, PCR: Polymerase chain reaction, IgG: Immunoglobulin G, AFB: Acid-fast bacilli, TB: Tuberculosis, VDRL: Venereal disease research laboratory, IgA: Immunoglobulin A, RPR: Rapid plasma regain.

Investigations of the first hospital visit (December 2019)			
WBC	4.2 10^9^/L	ANA	Negative
Hemoglobin	15.3 g/dL	C3/C4	1.07/0.23 g/L
PLT	292 10^9^/L	MOG antibodies	<1:10
Creatinine	73 µmol/L	AQP4 antibody	<1:10
ALT	28 U/L	IgG/IgA/IgM	13.8/1.44/0.65 g/L
ALP	43 U/L	ESR	12 mm/hr
Albumen	35.7 g/L	CRP	10 mg/L
TSH	1.490 µlU/mL	Blood & Urine Cultures	Negative
LDL	2.24 mmol/L	Brucella antibodies	Negative
Hemoglobin A1C	10.7%	Urethral swab for gonorrhea & chlamydia	Negative
Vitamin B-12	397 pmol/L	HBV surface antigen	Negative
25 Ho-Vitamin-D	63.1 nmol/L	HCV antibodies	Negative
Vitamin B1	169 nmol/L	4^th^ generation HIV antibody/antigen	Negative
Vitamin B6	66.0 nmol/L	TP-specific recombinant antigens (CLIA)	Positive
Celiac antibodies	Negative	TP-antibodies (EIA)	Positive
ACE level	24 U/L	TP – IgM (EIA)	>100 U/mL
CSF analysis of the first hospital visit (December 2019)			
CSF appearance	Colorless	CSF AFB smear	Negative
CSF WBC	136 CUMM (7%polymorph, 93%mononuclear)	CSF TB-PCR	Negative
CSF RBC	0 CUMM	CSF VDRL test	Negative
CSF protein	0.69 g/L	CSF oligoclonal bands	Not detected
CSF glucose	6.0 mmol/L	IgG/Albumen ratio	0.8
The BioFire ® FilmArray ® Meningitis/Encephalitis (ME) Panel	Negative for all tested pathogens	CSF bacterial culture	Negative
CSF IgG	0.108 g/L	CSF Albumen	0.367 g/L
Investigations of the second hospital visit (August 2020)			
WBC	2.8 10^9^/L	Procalcitonin	<0.02 ng/mL
Hemoglobin	16.1 g/dL	ESR	5 mm/hr
PLT	234 10^9^/L	CRP	3 mg/L
Hemoglobin A1C	6.7%	TP-specific recombinant antigens (CLIA)	Negative
ALT	29 U/L	RPR test from serum	1:1
Albumin	45 g/L	AQP4 antibody	<1:10
CSF analysis of the second hospital visit (August 2020)			
CSF appearance	Colorless	CSF protein	0.69 g/L
CSF WBC	0 CUMM	CSF glucose	6.0
Xanthochromia	Negative	Serum glucose	9.2 mmol/L
CSF RBC	0 CUMM	TP-PCR from CSF	Negative

**Figure 4:**
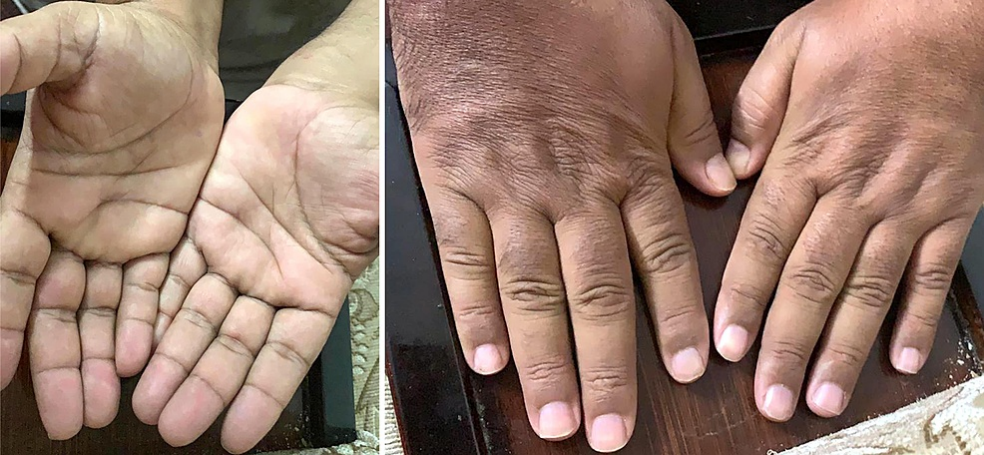
Clinical skin examination on the second episode. Hand palm and nails clinical examination during the second episode showing the absence of rashes or nail changes.

Unfortunately, due to the COVID-19 pandemic, he was reluctant to visit the hospital and presented to the hospital after two months. There were no rashes during his physical examination nor any new neurological symptoms (Figure 4). Brain MRI was repeated; it was unremarkable. This time, VEP revealed absent NPN complexes in both eyes.

**Figure 5:**
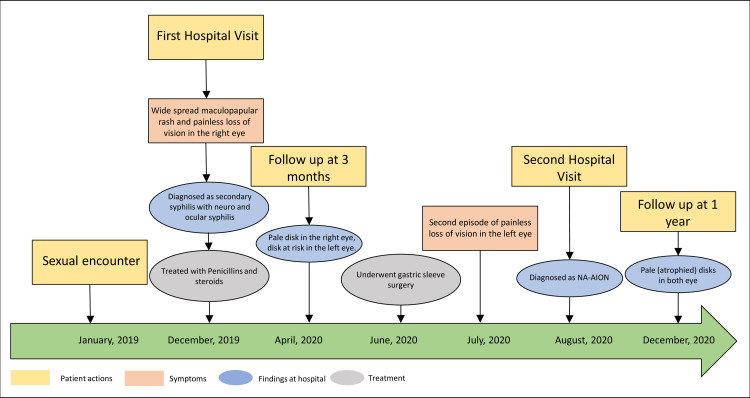
Timeline of events from sexual encounter till final follow-up at one year from the initial symptoms

A neuro-ophthalmologist evaluated the patient; the final diagnosis was NA-AION (Figure 5). Behavioral counseling and management of other controllable risk factors such as smoking and diabetes were explained to the patient to minimize/prevent disease progression.

## Discussion

*T. pallidum* invasion of the meninges is not uncommon, it was estimated at 0.47 to 2.1 cases per 100,000 population and potentially higher when the patient is human immunodeficiency virus (HIV) co-infected [6]. Although neurosyphilis is uncommon, the ocular subtype is increasingly reported, probably due to increased rates of syphilis in general [3]. The meningeal invasion happens within days and can manifest at any stage of the disease [6]. Neurosyphilis has to be ruled out in cases of ocular syphilis for multiple reasons, including confirming the diagnosis and establishing parameters for assessing the success of the given treatment [7].

Chancre is the hallmark of primary syphilis skin manifestation, which is a solitary painless indurated ulcer with a clean base and heaped-up margins that can appear at the site of contact with lesions of the sexual partner and not only at the genital areas, but multiple painful ulcers can also present [8]. While secondary syphilis has more diverse skin manifestations, including a ﻿non-pruritic maculopapular rash, especially on the palms and soles, lymphadenopathy, mucosal patches, condyloma lata, and alopecia [1,8]. Other rare skin manifestations of secondary syphilis include nail changes described as syphilitic onychia (changes on the nail plate) and syphilitic paronychia (changes in the periungual tissue) [9]. The five manifestations of syphilitic onychia include onychoptosis (periodic partial or complete shedding of the nail), onycholysis (detachment of the nail from its bed), onychomadesis (acute, non-inflammatory, painless proximal separation of the nail plate from the nail matrix due to temporary arrest of growth of the nail matrix), onychogryphosis (asymmetric growth of the nail sides), and nail pitting [9-11]. Onychomadesis has several other associations with drugs like some chemotherapeutic agents, azithromycin, and lithium; infections like hand-foot-mouth disease, varicella infection, and fungal infections; systemic diseases like Stevens-Johnson syndrome, toxic epidermal necrolysis, and lichen planus or other causes like nail trauma and idiopathic causes [11].

Direct detection of *T. pallidum* from the tissue by darkfield microscopy is the definitive diagnosis method in early syphilis [12]. On the other hand, there is no gold-standard test to confirm neurosyphilis or ocular syphilis, but several diagnostic tests can increase the result's yield and confidence [3,6]. Visual symptoms can be the initial presenting symptoms in cases of syphilis and have to be investigated thoroughly [13]. Most guidelines recommend establishing the diagnosis of ocular syphilis in suspected cases by first confirming testing for systemic syphilis using the traditional or the reverse sequence algorithms [3]. Once evidence of the disease is found, CSF should be tested for venereal disease research laboratory (VDRL), which, if positive, can be diagnostic (in the absence of blood contamination of the CSF) [12]. Negative VDRL warrants testing for Fluorescent Treponemal Antibody Absorption (FTA-ABS) test, total white blood cells, and proteins from the CSF [12]. All serological tests have alternatives that can be used in case of unavailability [13]. Elevated WBC count in CSF is suggestive of neurosyphilis, but the diagnostic cut-off can differ slightly in case of co-infection with HIV (20 mm^3^ in HIV co-infected patients while 5 mm^3^ in others) [12]. Careful interpretation should be applied in each case as ocular syphilis patients can have normal CSF examinations; abnormal CSF examinations can also happen in asymptomatic syphilis patients with possible progression to neurological complications after diagnosis [3].

The polymerase chain reaction test from the CSF has shown low sensitivity of 40%-70% with a very high specificity of 60%-100%, similar to VDRL testing from CSF which has low sensitivity with high specificity but unlike FTA-ABS from the CSF, which is very sensitive but unspecific [3]. MRI can assist in the diagnosis if the optic nerve was affected by showing enhancement in the fat-suppressed images [14,15] or enhancement of the meninges, cerebral atrophy, or changes consistent with infarction [16], but also can be normal [16,17]. FFA leakage and other types of hyper-fluorescence have been reported in the literature as an aid to support the diagnosis too [7,18,19], but they are non-specific [20]. In optical coherence tomography (OCT), no changes [19], or other complications like subretinal fluid collection and loss of thickness at the macula or retinal nerve fiber layer (RNFL) can be seen [14,18,21].

Misdiagnosing ocular syphilis as a non-infectious disease has been reported even when seen by ophthalmologists due to the wide variety of possible presentations [7]. In general, ON (a generic term for inflammatory optic neuropathies) is difficult to distinguish from other forms of optic neuropathies, especially in middle-aged patients who have a painless acute loss of vision with disc edema [16]. Ischemic optic neuropathy (ION), whether arteritic vs. non-arteritic, has been reported as either a misdiagnosis [19] or in a non-causal relationship with syphilis [22,23]. NA-AION has also been reported with Lyme disease (a disease caused by *Borrelia burgdorferi*, a spirochete like *T. pallidum*) [24]. Syphilis can arguably trigger or cause an immune-mediated optic neuropathy like seropositive and seronegative variants of neuromyelitis optica spectrum disorder (NMOSD), which has been reported in several articles with ocular and or neurosyphilis [25-28], or NMDAR antibody encephalitis [29]. Other differentials for painless loss of vision include Leber's hereditary ON (LHON) [14].

VEP can be used to detect the retina's loss of function throughout the optic nerve up to the visual cortex in the brain [30]. In ocular and/or neurosyphilis, VEP can present as an absence of waves, all types of latencies, changes in the morphological waveforms [31], or can be normal [32]. The test can also differentiate between ON and ION, mainly by the duration of latency of the P100 (prolonged in ON while mildly prolonged or normal in ION) [30,33]. Other findings include reduction in amplitude and morphological changes in the waveform [22,33,34].

Ocular syphilis should be treated as neurosyphilis, where an adequate and prolonged concentration of treponemicidal antibiotics should be given [3]. Hence, intravenous short-acting aqueous penicillin G is the preferred drug of choice since it can achieve the required concentrations [3]. Other guidelines suggest alternatives like intramuscular procaine penicillin G with oral probenecid, benzylpenicillin, or ceftriaxone [6]. Corticosteroid use for the treatment of ocular syphilis is controversial, yet some experts may recommend it to avoid Jarisch-Herxheimer’s reaction too [3]. Patients with NA-AION may also benefit from the use of corticosteroids which supports their use in similar cases [35]. Prognosis is unpredictable, some patients respond within weeks, while others have permanent damage and become legally blind [3]. It has been noticed that delayed diagnosis and delayed initiation of treatment has been linked to poor outcome [3].

Failure of treatment is difficult to assess, but generally, serum titers of non-treponemal tests should decrease fourfolds in six to 12 months [12]. For neurosyphilis, on the other hand, if CSF WBC did not decrease in six months or normalize along with CSF proteins by 24 months, re-treatment should be considered [12]. Other parameters of failure of treatment include a progression of neurological symptoms and an increase in the non-treponemal titers by more than two dilutions [3]. Serum Rapid Plasma Reagin (RPR) titers can predict CSF parameters' response and help avoid invasive testing [12].

In the presented case, ON was not considered in this patient as ophthalmological examination did not reveal any signs of inflammation at any part of the eye in the first and the second episodes. The presence of splinter hemorrhages with disc edema in the affected eye’s retina and disc at risk in the other eye is suggestive of NA-AION as well as the sequential presentation in both eyes. NMOSD has been ruled out since the patient's condition does not fit the clinical diagnostic criteria (the absence of typical symptoms, negative enhancement in the fat-suppressed MRI images of the brain and spine with negative AQP4 antibodies in both episodes), which has around 99% sensitivity and around 90% specificity [36]. OCT was unremarkable while VEP showed typical NA-AION features in the left eye (slight latency in the P100 at 109 ms and deformed wave with low amplitude) in the first episode and absent NPN complexes in both eyes after the second attack, suggesting damage to both optic nerves. Finally, lack of improvement in vision after a documented response to a proven neurosyphilis treatment (normalization of CSF WBC and protein + disappearance of syphilis antigens in screening test + RPR titers of 1:1) is highly suggestive of NA-AION rather than ON. To our knowledge, this is the first case in the literature to describe the potential causal relationship between ocular syphilis and NA-AION which has to be further studied to confirm the relationship and the pathophysiology behind it.

## Conclusions

Ocular syphilis is a rare disease with many presentations in the form of inflammation of almost any part of the eye. Immune-mediated inflammation can also be triggered by syphilis. However, to our knowledge, ION was not a known sequela of ocular syphilis. Out of all types of ION, NA-AION is a diagnosis of particular interest, as it may cause a great dilemma to the treating physician with many unanswered questions. Early proper diagnosis and treatment can cure ocular syphilis and improve vision in some cases. While, history of progressive, painless loss of vision in a patient diagnosed with syphilis that does not respond to appropriate treatment warrants further investigations to rule out immune-mediated and ischemic optic neuropathies that may be associated with or triggered by syphilis “The Great Mimicker.”
